# Lead-Free BiFeO_3_-Based Piezoelectrics: A Review of Controversial Issues and Current Research State

**DOI:** 10.3390/ma15134388

**Published:** 2022-06-21

**Authors:** Sangwook Kim, Hyunwook Nam, Ilkan Calisir

**Affiliations:** 1 Graduate School of Advanced Science and Engineering, Hiroshima University, Higashihiroshima 739-8526, Hiroshima, Japan; 2 Graduate Faculty of Interdisciplinary Research, University of Yamanashi, Kofu 400-8510, Yamanashi, Japan; 3 Department of Chemistry, University of Liverpool, Liverpool L69 7ZD, UK

**Keywords:** lead-free, BiFeO_3_-based, piezoelectrics, pseudo-cubic, heat-treatment

## Abstract

Lead-free electroceramics represent an emerging area of research that has the potential to enable new green advances in electronics. Research has mainly focused on the development of new piezoelectric materials for replacing lead containing oxides exhibiting superior electromechanical behavior. Lead-free BiFeO_3_-based materials are not only the promising candidates to replace lead-based materials but also show intriguing properties which may inspire innovative material design for the next generation of lead-free piezoceramics. This review aims to highlight the current state of research and overlooked aspects in lead-free BiFeO_3_-based ceramics, which could be insightful in elucidating certain controversial issues. Current strategies to reduce high conductivity, influence of chemical heterogeneity on both functional properties and crystal structure, effective heat treatment procedures, and the role of pseudo-cubic structures on the enhancement of piezoelectric properties are subjects of highlighted within this review as they have a significant impact on the quality of BiFeO_3_-based lead-free piezoelectrics (but are often disregarded).

## 1. Introduction

Electroceramics, materials used in electronics as crucial components of semiconductors, mobile communication, and displays, have played an important role in modern industrial development. Dielectrics, piezoelectrics, varistors, ion-conductive solid cells, and solar cells have all been employed as electrical components and materials [1,2]. Among the electroceramics, piezoelectric materials, account for approximately 35% [3] of the electroceramics market. High-performance piezoelectric materials convert electrical energy to mechanical strain through either piezoelectricity or electrostriction. They are desired for a wide range of specific applications such as micromotors, transducers, acoustic and prosthetic devices and are key materials for the future of devices requiring low-power or even self-power [4].

A quartz sonar device for ultrasonic submarine detectors was developed by Paul Langevin in 1917 during World War I [5]. This breakthrough paves the way for the development of powerful and fast piezoelectric materials. In the 20 years following World War II, piezoelectric research focused on finding new materials and improving the quality of devices. During this period, the classical piezoelectric materials barium titanate (BaTiO_3_, BT) and lead zirconium titanate (Pb(Zr,Ti)O_3_, PZT) were discovered [6].

The breadth of research accumulated over decades since 1950 have revealed vital piezoelectric properties and several mechanisms of lead-based piezoelectric materials. As a result, piezoelectric materials that have been commercially used thus far are lead-based (for example, PZT ceramics that exhibit excellent piezoelectric properties [7,8,9]. However, lead can cause environmental problems and human health related issues, and the European Union regulates the use of harmful substances, such as lead, in electronic devices [10]. Thus far, lead-free piezoelectric materials have not been developed yet. Therefore, lead-based piezoelectric ceramics are generally granted an exception. However, development of lead-free materials will lead to a removal of this exemption, with the overall aim of a completely lead-free industry in the future.

**Figure 1:**
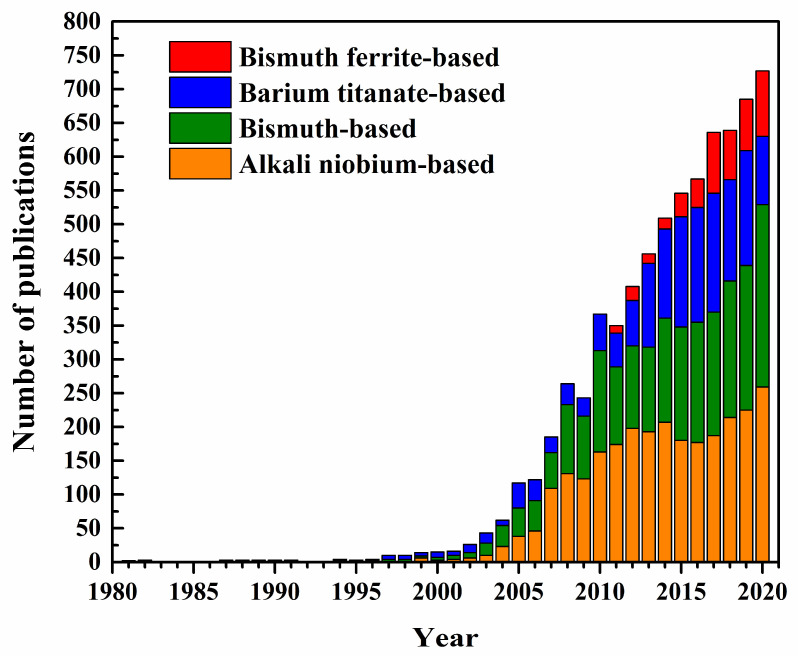
Evaluation of research output for lead-free piezoceramics over the last 40 years. Number of publications was investigated by searching for “piezoelectrics”, “lead-free”, and “piezoceramics” in Web of Science [11], each publication was manually checked for relevance.

Research on lead-free piezoelectric materials has rapidly increased since the early 2000s, as shown in Figure 1. New insights into bismuth-based perovskites have spawned new research fields in oxygen conductors, high-temperature dielectrics, and energy storage materials. In addition, with the recent development of bismuth ferrite (BiFeO_3_, BF)-based lead-free piezoelectric materials, the field has pioneered the development of high-temperature piezoelectric materials. The lead-free piezoelectrics are projected to grow even larger in the future.

Various lead-free piezoelectric materials have been studied, and representative lead-free piezoelectric ceramics can be broadly divided into four types: (Bi,Na)TiO_3_ (BNT)-based [12,13,14], (K,Na)NbO_3_ (KNN)-based [15,16,17], BaTiO_3_ (BT)-based [18,19,20], and BiFeO_3_ (BF)-based [21,22,23].The phase transition temperature and piezoelectric characteristics are the two most critical parameters for replacing lead-based piezoelectric materials. Therefore, numerous methods and mechanisms have been studied to improve the phase transition temperature or increase the piezoelectricity of lead-free piezoelectric materials to compete with the properties of lead-based piezoelectric materials. However, currently developed lead-free piezoelectric materials could intrinsically possess limitations such as low Curie temperature in BT-based systems. Therefore, novel processing methods, procedures, and mechanisms, such as cold sintering and domain engineering, are currently being developed. Among them, BF-based systems, which have recently caught attention as a replacement for lead-based piezoelectric materials, show intriguing mechanisms that have not fully been revealed and/or understood yet. BF-based systems are of particular interest to applications where high-temperature and piezoelectricity are simultaneously required.

In this review, research trends, proposed mechanisms, crystal structures, and issues that are reported in lead-free BF-based piezoelectric systems are summarized. In addition, future research directions and prospects are also discussed.

## 2. Lead-Free Piezoelectrics

**Figure 2:**
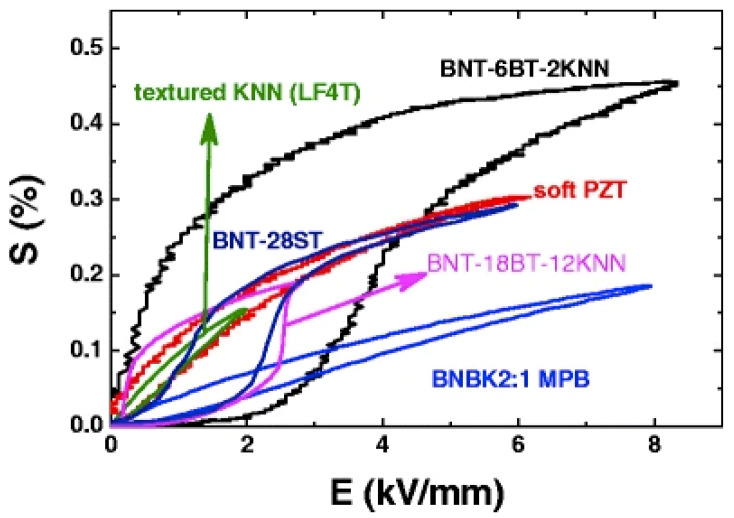
Electric-field-induced strain in comparison between soft PZT and BNT-based system. Reprinted with the permission of [28]. Copyright © 2012 Springer Nature.

Research on lead-free piezoelectric materials started in 1976, led by Professor Okazaki, resulting in (Bi,Na)TiO_3_–BaTiO_3_ (BNT–BT) systems in 1991 [24] and (Bi,Na)TiO_3_–(Bi,K)TiO_3_ (BNT–BKT) systems in 1999 [25]. Furthermore, a ferroelectric material with a bismuth-layered structure, which has been commercialized for resonators, was developed in the 2000s. Among them, it was reported that the BNT–BT–KNN ternary system [26,27] has a piezoelectric response comparable to that of the PZT system [28,29], as shown in Figure 2, which once again ignited research on the BNT-based system. In 2012, Jo et al. proposed that the large electric field-induced strain in lead-free BNT piezoelectric ceramics is due to the phase transition from the ergodic relaxor ferroelectric phase to the non-ergodic ferroelectric phase induced by external electric field stimuli, a phenomenon known as “incipient piezoelectrics” [28,30]. Despite the large electric-field-induced piezoelectric response, the required large electric field is a serious concern in BNT-based systems, since it would make it challenging to apply it towards actual devices.

**Figure 3:**
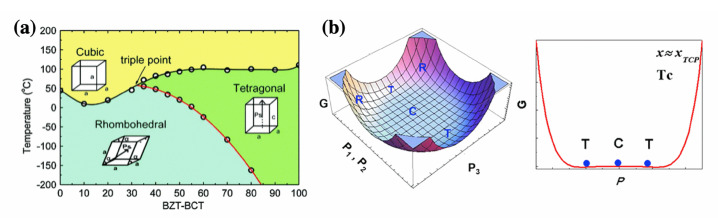
(**a**) Phase diagram of BZT–BCT system. Reprinted with the permission of [43]. Copyright © 2009 American Physical Society. (**b**) The (1$\bar{1}$0) projection of the free energy profiles for BZT–BCT system and 2D free energy profiles. Reprinted with the permission of [44]. Copyright © 2016 Elsevier.

BaTiO_3_, (BT) is a representative lead-free piezoelectric material that has been studied for a long time for the grain size effect [31,32,33,34], crystal structure at the atomic and electron level [35,36,37], nanoparticles [38,39,40], and texturing [41,42], and it is still being actively performed even to this day. Recently, Liu and Ren et al. suggested in 2009 that Ca and Zr co-doped BT (BZT–BCT) has the potential to replace the PZT system [43]. BZT–BCT has a tricritical point as the coexistence of crystal structures with cubic, tetragonal, and rhombohedral structures, as shown in Figure 3a, and exhibits the ultrahigh piezoelectricity of 620 pC/N [43]. As illustrated in Figure 3b, the energy profile near the triple point, with its flat free-energy landscape and low-energy barriers, favors polarization rotation. Thus, vanishing polarization anisotropy is the primary mechanism underlying the ultra-high piezoelectricity in Ba(Zr_0.2_Ti_0.8_)O_3_–(Ba_0.7_Ca_0.3_)TiO_3_ (BZT–BCT) ceramics [44]. Despite the high piezoelectricity of BT-based lead-free piezoelectric systems, their low Curie temperature (*T*_C_) < 130 °C could be seen as a limiting factor to their implementation in real devices [45].

**Figure 4:**
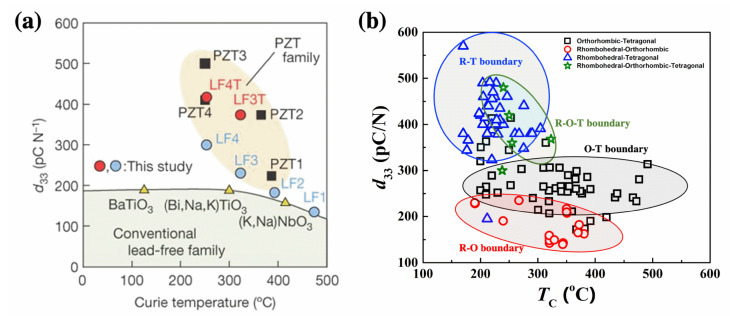
Piezoelectric coefficient *d*_33_ versus Curie temperature. (**a**) Comparison between the developed (K_0.44_Na_0.52_Li_0.04_)(Nb_0.86_Ta_0.10_Sb_0.04_)O_3_ (LF) ceramics and the conventional PZT ceramics. Reprinted with the permission of [46]. Copyright © 2004 Springer Nature. (**b**) Comparison according to type of phase boundary in KNN-based ceramics.

Y. Saito et al. reported in 2004 that textured KNN systems have a strong piezoelectric property [46], as shown in Figure 4a. The KNN-based system features a polymorphic phase boundary (PPB) in which the phase transition depends on the temperature [47,48,49], and strategies to improve piezoelectric properties by forming a PPB near room temperature are a major issue in KNN-based piezoelectric systems.

The PPB contains a phase transition boundary from orthorhombic to tetragonal (O-T) [50,51,52,53,54], rhombohedral to tetragonal (R-T) [55,56,57,58,59,60,61], and rhombohedral-orthorhombic-tetragonal (R-O-T) boundary [62,63], and each of which exhibited a significant change in piezoelectricity depending on the phase boundary, as shown in Figure 4b. At the early stages of the research on PPB in KNN-based systems, research was focused on orthorhombic to tetragonal (O-T) phase boundary. Later, rhombohedral-to-orthorhombic (R-O) phase boundaries were investigated in detail. Recently, PPB with rhombohedral to tetragonal (R-T) phase boundaries has been extensively studied, and it has been reported in the literature that it yields a larger piezoelectric response compared to other PPB-type phase boundaries in KNN-based systems. The piezoelectric properties of KNN-based ceramics with an R-T phase boundary were significantly improved to over 400 pC/N [56]. However, there is a major issue that *T*_C_ drops to approximately 240 °C as its piezoelectric response is maximized. Therefore, in recent years, a considerable effort has been expended to design a material composition with a high Curie temperature and an R-T phase boundary in a KNN-based system.

**Figure 5:**
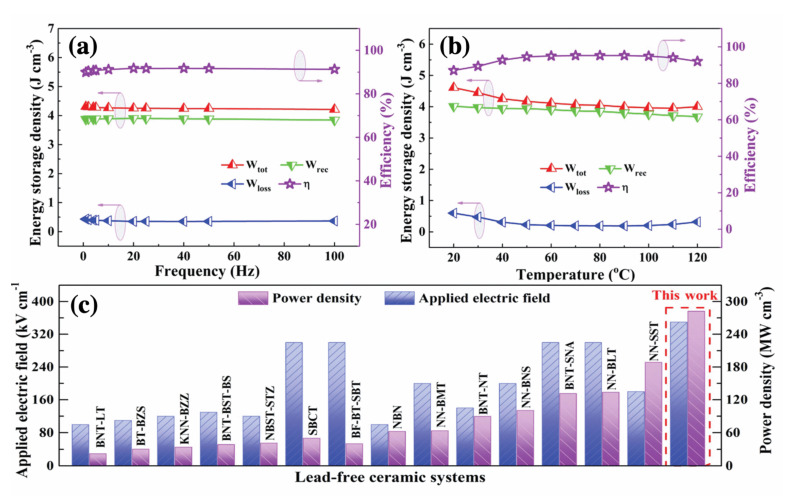
Energy storage performance dependent (**a**) frequency, (**b**) temperature for 0.35BiFeO_3_–0.65SrTiO_3_ ceramics. (**c**) The power density and applied electric field between other lead-free system and 0.35BiFeO_3_–0.65SrTiO_3_ ceramics. Reprinted with the permission of [65]. Copyright © 2022 John Wiley and Sons.

In view of these technological breakthroughs and constraints in lead-free piezoelectric solid solution systems, BF-based systems have been studied to reveal its potential to be used in applications requiring particularly high temperature and stable piezoelectric response. Lead-free BF-based ceramics have been studied with various composition such as BF–SrTiO_3_ (BF–ST), BNT–BF, BNT–BKT–BF, etc. [64,65,66,67,68,69]. BF–ST is being studied for energy storage application. Recently, Yan et al. reported the ultrahigh energy density of 9.3 J/cm^3^ and excellent efficient with 90% as shown in Figure 5a,b, which is an excellent energy density property compared to the that of other lead-free system, as shown in Figure 5c [65].

**Figure 6:**
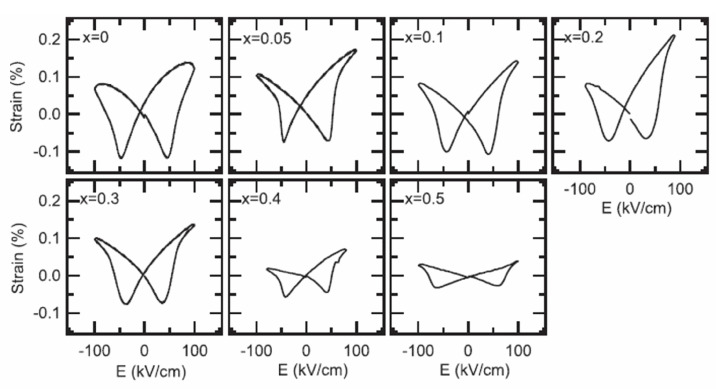
Bipolar piezoelectric response of (1−*x*)(Bi_0.5_Na_0.5_)TiO_3_–*x*BiFeO_3_ ceramics. Reprinted with the permission of [69]. Copyright © 2016, The Materials Research Society.

The piezoelectric properties were evaluated in various BF-based systems. The piezoelectricity was 69 pC/N in 0.625BF–0.375ST [64]. However, BF–ST system exhibited the instability of piezoelectricity with time [64]. Fujii et al. reported the piezoelectricity of a BNT–BF system, as shown in Figure 6 [69]. The large signal piezoelectric response (*d*_33_^*^) exhibited from 32 to 182 pm/V as a function of BF concentration. However, an asymmetry piezoelectric response was exhibited in wide range of composition due to a strong internal bias by defect dipoles, as shown in Figure 6, which might be a hindrance to apply for applications.

In 2015, Lee et al. reported the high piezoelectric performance (402 pC/N) of a BiFeO_3_–BaTiO_3_ (BF–BT)-based system [70], which led to various studies and developments on lead-free BF-BT-based piezoelectric systems. BF–BT-based systems have drawn attention as the next generation of lead-free piezoelectric systems. The recent advances in BF-based systems will be reviewed, and future research directions and prospects will be described in the next section.

## 3. (BiFeO_3_–BaTiO_3_)-Based Lead-Free Piezoelectrics

BF is gaining interest as a multiferroic material exhibiting ferromagnetic properties and ferroelectricity at ambient temperature. In addition, the *T*_C_ of BF is as high as 825 °C [71]. However, its piezoelectric properties are poor, which makes it difficult to use as a potential lead-free piezoelectric material. Recently, a BF–BT system combining the high piezoelectricity of BT and high *T*_C_ of BF has been proposed to replace lead-based piezoelectric systems [72,73]. The BF–BT and BF–BT-based lead-free piezoelectric systems will be the focus in the following sections.

### 3.1. Crystal and Domain Structures

**Figure 7:**
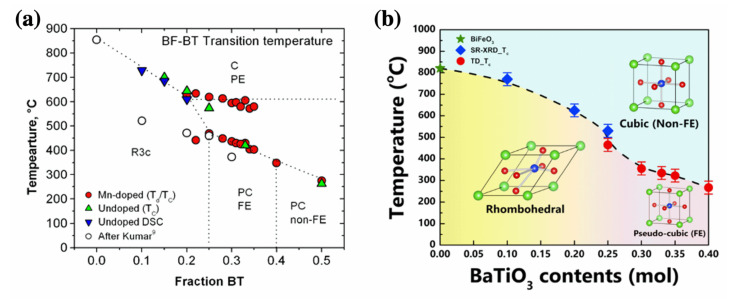
Phase diagram of BiFeO_3_-BaTiO_3_ system; (**a**) reported by Eitel. Reprinted with the permission of [75]. Copyright © 2009 John Wiley and Sons. (**b**) Reported by Kim. Reprinted with the permission of [72]. Copyright © 2017 AIP Publishing.

**Figure 8:**
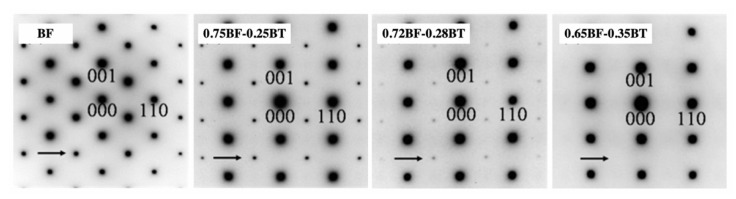
Electron diffraction patterns at room temperature with [1$\bar{1}$0] incidence in BF–BT ceramics. Reprinted with the permission of [76]. Copyright © 2008 Taylor and Francis.

In 1981, Ismailzada et al. reported that three crystal structures were assigned to BF–BT solid solution systems depending on the mol% of BF content at room temperature as following 100–67 mol% of BF: rhombohedral, 67–7.5 mol% of BF: cubic, and 7.5–0 mol% of BF: tetragonal [73]. Subsequently, the phase equilibrium diagram of BF–BT was proposed by Kumar in 2000 [74]. In 2009, Eitel et al. suggested a revised phase diagram of the BF–BT system, as shown in Figure 7a [75], which was illustrated based on the evidence of the temperature dependence of the dielectric constant and differential scanning calorimetry (DSC). Eitel et al. suggested that the crystal structure of (1-*x*)BF–*x*BT is rhombohedral with an *R*3*c* space group of up to *x* = 0.25 and a pseudo-cubic structure between *x* = 0.25 and *x* = 0.40. Above *x* = 0.40, the crystal structure transforms into a cubic structure. Subsequently, Lee et al. suggested a morphotropic phase boundary (MPB) with rhombohedral and tetragonal structures using X-ray diffraction (XRD) [70]. Similar to PZT ceramics, MPB with rhombohedral and tetragonal structures were formed in the *x* = 0.33 composition. In 2017, Kim et al. investigated the crystal structure of the BF–BT system using high-energy synchrotron radiation XRD as a function of temperature, and suggested a new phase diagram of the BF–BT system, as shown in Figure 7b [72]. The crystal structure at *x* = 0.25 is found to be similar to a rhombohedral structure of BF. The crystal structure undergoes a transition from rhombohedral to pseudo-cubic at the boundary between *x* = 0.25 and *x* = 0.30 compositions. Kitagawa et al. examined the crystal structure by electron diffraction; the rhombohedral to cubic structure transformation at the boundary between *x* = 0.28 and *x* = 0.35 was observed with the disappearance of superlattice reflection peaks (as shown in Figure 8) [76], which could be supported by the phase diagram suggested by Kim [72].

**Figure 9:**
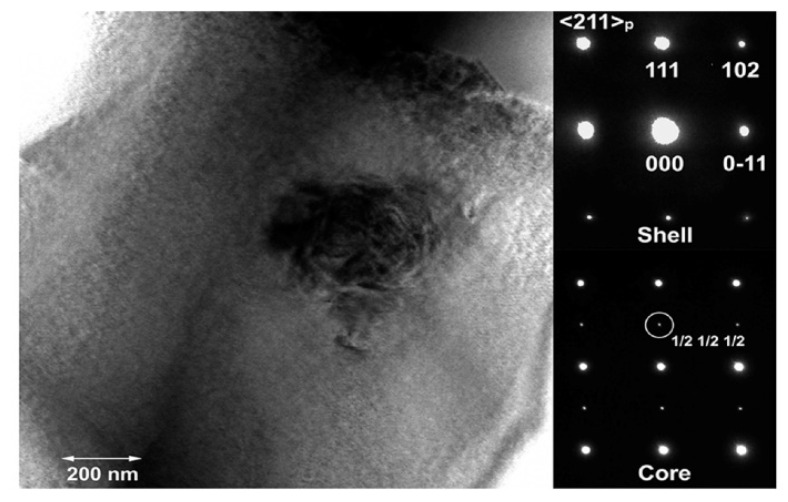
Bright field TEM image of 0.05Bi(Zn_2/3_Nb_1/3_)O_3_-doped BF–BT, and diffraction pattern in core and shell regions. Reprinted with the permission of [78]. Copyright © 2018 American Chemical Society.

Recently, Calisir et al. observed a core-shell structure in 0.75Bi(Fe_0.99_Ti_0.01_)O_3_–0.25BaTiO_3_ lead-free piezoelectric ceramics. A high-energy synchrotron X-ray diffraction method was utilized to determine the crystal structure of core and shell phases in this solid solution system [77]. In addition, they suggested that the crystal structures of the core and shell parts within grains were altered by the application of various heat treatments (slow cooling or quenching). Both structures of the core were rhombohedral with the *R*3*c* space group. However, the crystal structure of the shell is found to be pseudo-cubic structure (*Pm*$\bar{3}$*m*) in slow-cooled 0.75Bi(Fe_0.99_Ti_0.01_)O_3_–0.25BaTiO_3_ ceramics and rhombohedral structure (*R*3*m*) in quenched 0.75Bi(Fe_0.99_Ti_0.01_)O_3_–0.25BaTiO_3_ ceramics. The formation of a core-shell structure, namely chemically heterogenous grains, has also been reported in other BF–BT-based materials. For example, Wang et al. showed the core-shell structure of Bi(Zn_2/3_Nb_1/3_)O_3_-doped BF–BT [78] lead-free system. They reported that {1/2 1/2 1/2} superlattice reflections in the *R*3*c* space group were observed in the BF-rich core region, whereas the BT-rich shell consists of a cubic structure with a nano-domain, as shown in Figure 9.

One issue with the crystal structure in BF-based lead-free piezoelectric materials is the presence of pseudo-cubic structure. Various BF-based lead-free systems with pseudo-cubic structure exhibit significantly large piezoelectric response and ferroelectricity [62,79,80,81]. Various detailed crystal structure refinement methods, precise techniques, and associated mechanisms have been proposed to clarify the accurate crystal structure and underlying mechanism of such enhancement of ferroelectricity and piezoelectric activity in the vicinity of pseudo-cubic phase in BF-based lead-free systems. The detailed mechanism and the crystal structure of BF-based lead-free system are described in the next section.

### 3.2. Ferroelectric Domain Structure

The domain configuration of piezoelectric materials can determine the electrical properties, including dielectric, ferroelectricity, pyroelectricity, and piezoelectricity. Controlling the domain configuration to modify the piezoelectricity of a material is called domain engineering, and is an interesting topic in piezoelectric materials. Numerous strategies for domain engineering have been proposed, including texturing techniques, single crystal growth, application of electric field and compositional design [82,83,84,85,86,87,88,89]. In particular, piezoelectricity can be controlled by inhibiting or enhancing the domain wall motion by doping, donor or acceptor doping in PZT ceramics, for example [89]. Recently, it was found that the piezoelectricity in BT has improved dramatically as the domain wall density increased owing to domain size reduction [90,91]. The ferroelectric domains play an important role in the piezoelectricity of piezoelectric ceramics; therefore, investigating the domain structure is important for understanding ferroelectricity and piezoelectricity.

**Figure 10:**
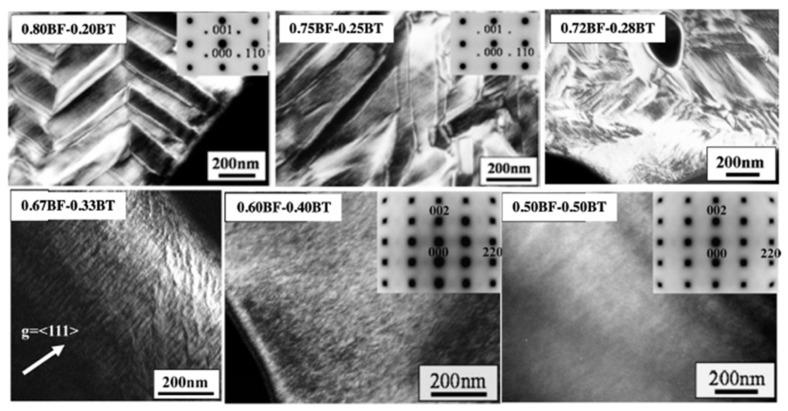
Domain structure of BiFeO_3_–BaTiO_3_ system as a function of BaTiO_3_ contents. Reprinted with the permission of [76]. Copyright © 2008 Taylor & Francis. Reprinted with the permission of [92]. Copyright © 2009 Taylor & Francis.

The domain structure was evaluated as a function of the chemical composition, temperature, and electric field in order to understand the ferroelectricity and piezoelectricity in BF-based lead-free system. Recently, Mori et al. evaluated the domain structure in BF–BT solid solution system [76,92]. The lamellar domain structure was found in 0.80BF–0.20BT, and changed to the herringbone-type domain structure in 0.72BF–0.28BT, as shown in Figure 10. A tweed-like domain structure was shown in 0.67BF–0.33BT and a nanosized domain structure was observed in the composition above 0.60BF–0.40BT ceramics. The maximum piezoelectricity was exhibited at the phase boundary where rhombohedral and cubic phases are coexisted with the presence of predominant nanosized domain structures.

**Figure 11:**
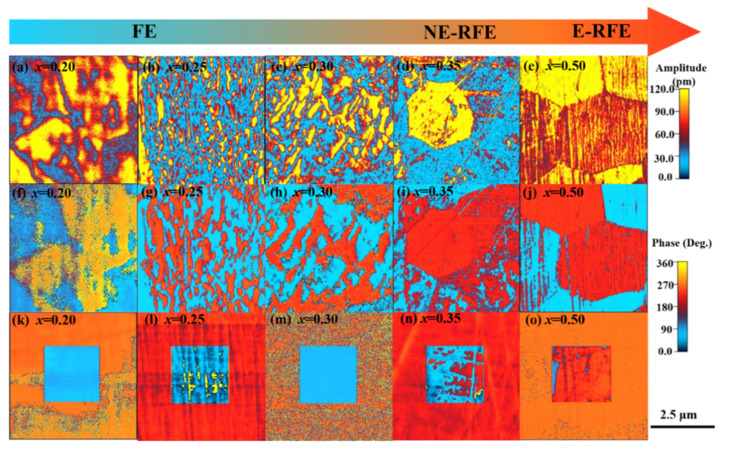
PFM scanning image of (1-*x*)BiFeO_3_–(*x*)BaTiO_3_. Out-of-plan amplitude; (**a**) *x* = 0.20, (**b**) *x* = 0.25, (**c**) *x* = 0.30, (**d**) *x* = 0.35, and (**e**) *x* = 0.50. Out-of-plan phase; (**f**) *x* = 0.20, (**g**) *x* = 0.25, (**h**) *x* = 0.30, (**i**) *x* = 0.35, and (**j**) *x* = 0.50. The “box-in-box” DC writing under the ±10 V; (**k**) *x* = 0.20, (**l**) *x* = 0.25, (**m**) *x* = 0.30, (**n**) *x* = 0.35, and (**o**) *x* = 0.50. Reprinted with the permission of [93]. Copyright © 2018 American Chemical Society.

The domain structure change in size, macro-sized domain to nano-sized domain in a polar nano region in the BF–BT system in phase across rhombohedral to pseudo-cubic was reaffirmed by piezoresponse force microscopy (PFM), which is a useful technique for determining the domain structure. Xun et al. assessed the domain structure in BF–BT system, as shown in Figure 11 [93]. A stripe-type macro-sized domain structure with rhombohedral symmetry was shown in 0.80BF–0.20BT. The density of domain walls in 0.75BF–0.25BT was increased as the domain size is reduced. As the amount of BT increased, the domain size decreased, and the striped type of domain structure in 0.70BF–0.30BT consists of blocks and island-like structures. The 0.65BF–0.35BT ceramic exhibits large areas in a variety of colors, representing the shape of microscopic areas, which are comprised of island-like domains and polar nano regions (PNRs) which are characteristics of relaxor type ferroelectrics. The domain size change according to the composition of the BF–BT system suggests that domain engineering is an applicable strategy in BF–BT-based systems.

**Figure 12:**
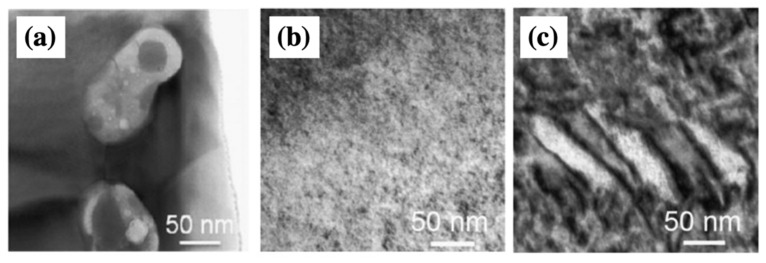
Bright-field TEM image of domain structure; (**a**) 0.40BaTiO_3_–0.60Bi(Mg_1/2_Ti_1/2_)O_3_, (**b**) 0.45BaTiO_3_–0.20Bi(Mg_1/2_Ti_1/2_)O_3_–0.35BiFeO_3_, and (**c**) 0.30BaTiO_3_–0.10Bi(Mg_1/2_Ti_1/2_)O_3_–0.60BiFeO_3_ ceramics. Schemes follow the same formatting. Reprinted with the permission of [94]. Copyright © 2012 Elsevier.

Recently, Wada et al. applied domain engineering with compositional design in a Bi(Mg_1/2_Ti_1/2_)O_3_-doped BF–BT (BT–BMT–BF) system as a function of BF concentration [94]. The domain size was controlled by the BF content, and decreased as the BF content reduced, as shown in Figure 12. The macro-and nano-sized domains coexist in the 0.30BT–0.10BMT–0.60BF composition, as shown in Figure 12c. The macro-domain patterns disappeared in the 0.45BT–0.20BMT–0.35BF composition, and only the nanosized domains remained. In contrast to the BT-based ceramics, ferroelectricity and piezoelectricity in the BT–BMT–BF system decreased due to the reduced domain size. Wada et al. suggested that piezoelectricity and ferroelectricity improved as the domain wall density increased owing to the coexistence of nano and macro-sized domains during the transition from ferroelectric to relaxor ferroelectric [94], which is called nano/macro-domain engineering. However, the mechanism of domain engineering with nano/macro-domains in BF-based systems is not conclusive yet. Various studies have been conducted to understand domain engineering in BF-based lead-free piezoelectric materials. However, domain engineering research for BF-based systems is still in its infancy. As a result, it is anticipated that domain engineering will play a critical role in increasing piezoelectricity, and several studies utilizing domain engineering will be undertaken.

### 3.3. Piezoelectric Properties

The BF–BT system has a relatively high *T*_C_, and the variation in *T*_C_ and static piezoelectricity as a function of BF–BT ratio have attracted attention for application in various devices. The piezoelectric coefficient, *d*_33_ of BF–BT system with *T*_C_ is 109.33 pC/N for 0.80BF–0.20BT with 625 °C, 137.26 pC/N for 0.75BF–0.25BT with 465 °C^,^, 79.15 pC/N for 0.70BF–0.30BT with 356 °C, 70.39 pC/N for 0.67BF–0.33BT with 334 °C, 56.24 pC/N for 0.65BF–0.35BT with 323 °C, and 33.81 pC/N for 0.60BF–0.40BT with 267 °C, respectively [72]. Despite the high *T*_C_ of the BF–BT system, the piezoelectric properties are approximately similar to those of other Bi-based lead-free piezoelectrics, such as BNT–BT. Therefore, various methods have been employed to improve the piezoelectric properties, but various issues, such as high conductivity, impede the improvement of the piezoelectric properties of the BF–BT system. Several strategies have been suggested to overcome these issues.

Doping and forming ternary systems are an established strategy to tune the piezoelectric properties of lead-free piezoelectric materials. Thus, to improve the piezoelectricity of the BF–BT system, methods, such as doping or creating ternary system have been introduced, and the majority of current research on BF–BT-based topic is populated around this method. Among the dopants in the BF–BT system, the successful ternary dopants are Bi(Mg_1/2_Ti_1/2_), Bi(Zn_1/2_Ti_1/2_), BiGaO_3_, Bi(Ni_1/2_Ti_1/2_), Bi(Mg_2/3_Nb_1/3_), and Bi(Zn_2/3_Nb_1/3_) [95,96,97,98,99]. These ternary dopants with BF–BT systems achieved a piezoelectric coefficient, *d*_33_ of approximately 150 pC/N and over 400 °C of Curie temperature, *T*_C_.

Recently, quenching has provided a new paradigm for improving the piezoelectric properties of lead-free BF-based systems. Lee et al. reported the highest piezoelectric coefficients of 324, 402, and 454 pC/N by water-quenching BF–BT with Bi(Zn_1/2_Ti_1/2_) and BiGaO_3_ doped systems, respectively [70,100]. BF–BT-based ceramics is significantly enhanced by the quenching or rapid cooling process compared to the non-quenching process (commonly referred as furnace cooling, slow cooling, as-sintered). Therefore, a quenching process is found to be one of the successful methods to improve piezoelectricity in BF-based lead-free systems, and this has led to further research on the effect of quenching processes, such as quenching temperature or cooling rate [101,102].

To evaluate piezoelectricity, a poling process is essential for piezoelectric ceramics. The piezoelectric coefficient and phase angle were measured after the direct current poling (DCP) process. However, a few reports have focused on the phase angle, which can provide important information as a measure of whether the completed domain switching by the poling process is carried out properly [103]. Recently, ultrahigh dielectric and piezoelectric properties in relaxor-PT crystals have been achieved by alternating current poling (ACP) rather than the conventional DCP process [104,105]. A study has been conducted to improve not only the phase angle but also the piezoelectric coefficient in the BF-BT system by combining the ACP and DCP processes [103]. However, this is a preliminary stage, and systematic ACP research is necessary to simultaneously monitor piezoelectric activity and phase angle.

**Figure 13:**
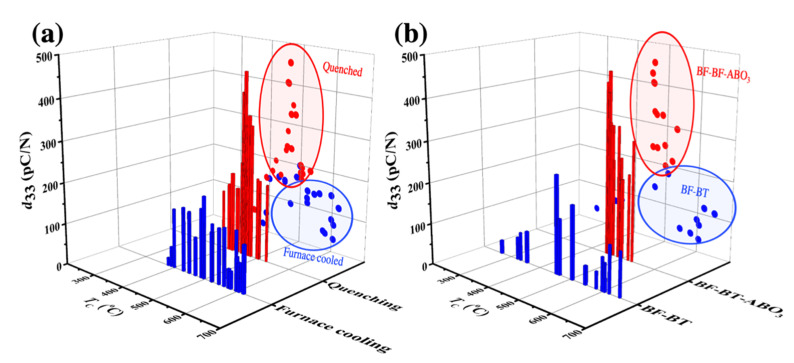
Piezoelectric coefficient *d*_33_ versus Curie temperature in BF–BT-based system. (**a**) Comparison between quenched ceramics and furnace cooled ceramics. (**b**) Comparison between BF–BT system and BF–BT–*AB*O_3_ system [70,75,78,97,98,99,106,107,108,109,110,111,112,113,114,115,116,117,118,119,120,121,122].

The improved piezoelectric properties of BF–BT-based systems have been studied via various approaches, such as doping and quenching. As illustrated in Figure 13a, the quenched BF–BT system exhibits superior piezoelectricity compared to that of the furnace-cooled BF–BT system. Also, the ternary systems, BF–BT–*ABO*_3_ show higher piezoelectric activity compared to the binary of BF–BT system, as shown in Figure 13b. The piezoelectric properties of BF–BT-based lead-free piezoelectric ceramics have been studied through various strategies, as described before, and piezoelectric properties have been improved by special methods, such as quenching or ternary systems; however, the origin of the enhancement and associated mechanisms are yet to be fully understood. Moreover, since excellent piezoelectric properties are exhibited in the BF–BT-based system in the region of the composition with a pseudo-cubic structure, research has been intensively conducted in the vicinity of the composition with a pseudo-cubic structure. However, the origin of piezoelectricity in pseudo-cubic structures remains uncertain. As a result, BF–BT-based lead-free piezoelectric ceramics have several controversies, such as structural disputes over the pseudo-cubic structure and quenching. The current controversial issues in BF–BT-based lead-free systems, such as the origin of piezoelectricity by doping or ternary systems and piezoelectricity in the pseudo-cubic structure, will be addressed in the next section.

### 3.4. Issues in BF–BT-Based System

#### 3.4.1. High Conductivity

**Figure 14:**
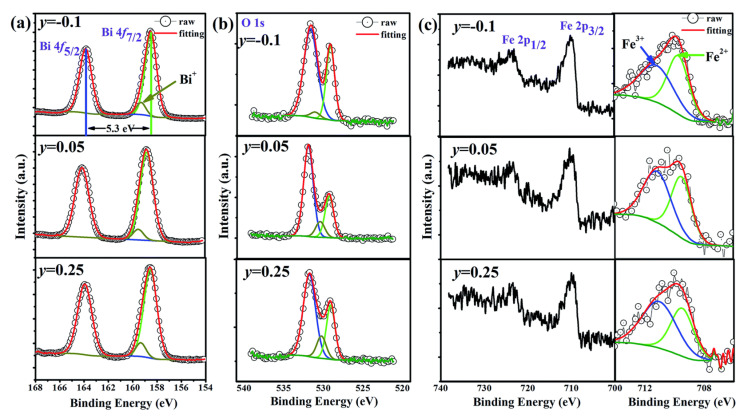
Valence states of the elements in (1−*x*)Bi_1+y_FeO_3+3y/2_–*x*BaTiO_3_ ceramics with *y* = −0.1, 0.05, and 0.25. (**a**) Bi 4f, (**b**) O 1s, and (**c**) Fe 2p. Reproduced from [126] with permission from the Royal Society of Chemistry.

The high conductivity hinders the improvement of piezoelectricity and ferroelectricity in BF-based systems. Cation and anion-charged defects due to the volatilization of Bi^3+^, oxygen vacancies, and the valence charge of Fe^3+^ in BF-based systems have been identified as causes of high conductivity [123,124]. These charged defects form a domain wall conduction. Recently, Rojac et al. determined the domain wall density in a polycrystalline BF [124,125]. The local domain wall conductivity disrupts the dynamics of the domain wall, which has unexpected effects on the piezoelectricity. Therefore, it has been suggested that the domain wall conductivity due to charged defects should be considered to evaluate the electromechanical properties [125]. Various methods, such as excess Bi or substitution of certain type of ions, have been suggested [126,127,128,129]. Recently, Wu et al. examined the concentration of oxygen vacancies and Fe^2+^ in a BF–BT system with Bi nonstoichiometric by XPS [126], as shown in Figure 14. The peak located at a binding energy of 529 eV is indicated by cation-oxygen bonds, and the other peaks located at 530 eV and 532 eV are absorbed by the surface H_2_O and oxygen vacancies. The concentration of oxygen vacancies decreased from 64% to 59.6% with increasing Bi content. However, the reduction was negligible. However, the valence of Fe^2+^ as a function of Bi content is distinct. The concentration of Fe^2+^ dramatically decreased from 49.5% to 40.4%, which enhanced both ferroelectricity and piezoelectricity.

**Figure 15:**
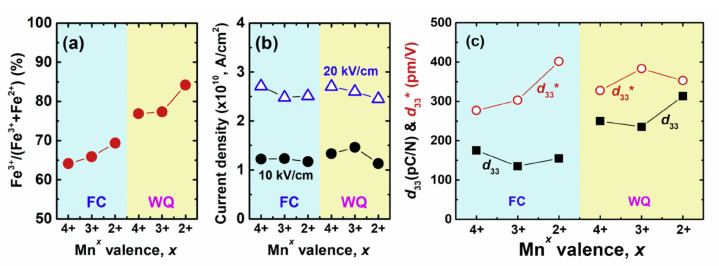
(**a**) Ratio of Fe^3+^, (**b**) Leakage current density, and (**c**) piezoelectric coefficients *d*_33_ and *d*_33_^*^ of the 0.67Bi_1.05_(Fe_0.99_Mn*^x^*_0.01_)O_3_–0.33BaTiO_3_ (*x* = 4^+^, 3^+^, and 2^+^) ceramics. Reprinted with the permission of [130]. Copyright © 2019 Elsevier.

Donor (high valence) and acceptor (low valence) dopants are used to suppress charged defects in BF-based systems. Song et al. investigated the donor and acceptor effects in a BF–BT system with multi-valent Mn (Mn^2+^, Mn^3+^, and Mn^4+^) substituted for Fe^3+^ [130]. The concentration of Fe^2+^ decreased due to acceptor Mn^2+^ doping with the furnace cooled BF–BT system in comparison with the donor Mn^4+^ doping, as shown in Figure 15, resulting in a decrease in the leakage current density with Mn^2+^ doping, as shown in Figure 15b. Note that the suppression of the Fe^2+^ transition is further strengthened by water-quenching, as shown in Figure 15a. The concentration of Fe^2+^ was significantly reduced by the quenching process, which induced a significant increase in the piezoelectric coefficient, as shown in Figure 15c. In general, the quenching process has a significant effect on the improvement of piezoelectric properties in BF-based systems, which may be due to the reduction of defects owing to the quenching process. However, it is still unclear why the defects are decreased by the quenching process. In addition, some researchers have confirmed that the piezoelectric and ferroelectric properties were improved even when quenching was performed after annealing at high temperatures. It cannot be argued that the quenching process reduces the number of defects since defects could be further increased during the annealing process at high temperatures. Therefore, quenching is an interesting research topic. Recently, various studies and proposals for understanding the quenching effect in BF-based systems have been reported. The topic of heat treatment (annealing or quenching) will be discussed in further depth in the following section (Section 3.4.3).

#### 3.4.2. Chemical/Compositional Inhomogeneity

**Figure 16:**
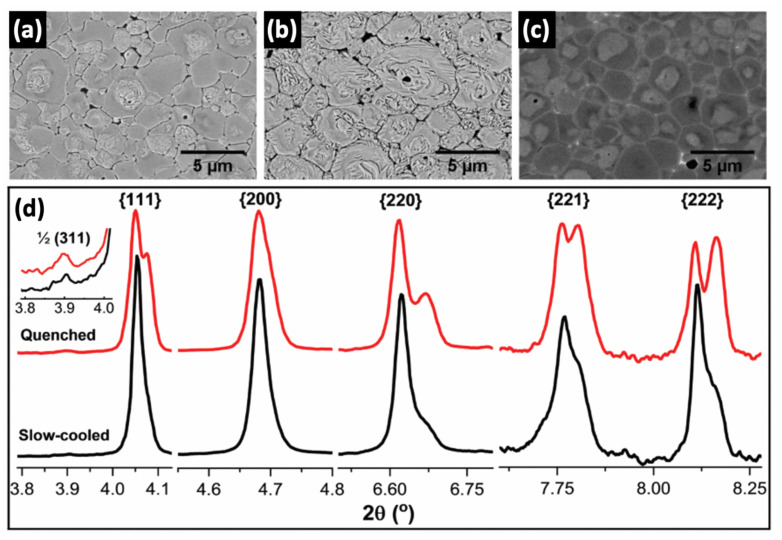
SEM images and high-energy XRD patterns of the 1 mol% Ti-doped 0.75BF–0.25BT ceramics. Chemically-etched surfaces of (**a**) slow-cooled and (**b**) quenched ceramics, and (**c**) non-etched polished surface of the quenched ceramic. (**d**) High-energy XRD profiles of slow-cooled and quenched ceramics for given crystallographic orientations. Reproduced from [77] with permission from the Royal Society of Chemistry.

Chemical homogeneity is a significant factor affecting the quality of a ferroelectric/piezoelectric ceramics. The chemical heterogeneity is often deliberately introduced to obtain a temperature and voltage stable dielectric ceramics, particularly it has widely and historically been employed in BT-based multilayer ceramics capacitors. Recently, the chemical heterogeneity has shown intriguing findings in BF–BT ceramics. This is often evidenced by a core-shell type microstructure composed of dark and light contrast relating to Ba/Ti-enriched and Bi/Fe-enriched regions shown in SEM images when backscattered electron mode is used. These regions are supposed to be composed of immiscible ferroelectric phases of BF and BT [77,97,131,132]. The use of certain types of dopants in BF–BT has been shown to cause chemical segregation and more intriguingly, it has been shown that donor types of dopants and certain *AB*O_3_-perovskite end-members could indeed promote chemical heterogeneity, while isovalent dopants promote solubility [77,97,131,132]. Murakami et al. [97,98] observed compositional inhomogeneity in Bi(*X*)O_3_-doped BF–BT, specifically when ‘*X*’ is selected as Y, Ga, Al, Sc_1/2_Y_1/2_, Mg_2/3_Nb_1/3_, Sc, Zn_2/3_Nb_1/3,_ and Zn_1/2_Ti_1/2_. The tendency on the observed thermodynamic immiscibility is suggested to be attributed to the electronegativity difference and covalency of dopants in BF–BT-based ceramics. This finding was also supported by the earlier work of Calisir et al., [132] as reported that the La substitution in Bi and Ba depending on the sites can indeed alter the electronegativity and covalency of the ion pair. In disagreement with the findings of Murakami et al. [98] quenching reduces heterogeneity, it was found that the core-shell microstructure was maintained with quenching, as evidenced by SEM images given in Figure 16a–c. A high-energy synchrotron XRD technique was used to examine the remarkable change in crystal structure in comparison to furnace (slow) cooled and quenched samples. After the application of quenching, broad single peaks in the furnace (slow) cooled ceramics indicating the presence of heterogeneous phases with higher fraction of pseudo-cubic phases transformed into multiple peaks, particularly at the characteristic peaks of rhombohedral phase {111} and {222} as shown in Figure 16d. Thus, one of the major issues observed in BF–BT-based ceramics is that the detailed microstructure examination in terms of determination of chemical heterogeneity is overlooked and this often leads to the misinterpretation of structure-property relations. The common observation of pseudo-cubic phase along with the rhombohedral phase in doped BF–BT ceramics could likely be associated with the presence of chemical heterogeneity as shown in Figure 16d. Therefore, the enhancement in the functional properties attributed to the presence of pseudo-cubic phase of BF–BT-based ceramics must be carefully evaluated by taking chemical homogeneity into account.

To control and/or eliminate the chemical heterogeneity in BF–BT ceramics, several approaches have been proposed. One is adding a small amount of MnO_2_ before the calcination [131] and the other is the use of isovalent dopants in substitution studies [64,97]. Chemical heterogeneity is not only often reported in BF–BT systems but also in BF–ST ceramics. Makarovic et al. [64] managed to eliminate the chemical segregation by employing high-energy ball milling also referred to as mechanochemical activated synthesis. Therefore, this method could also be utilized in the processing of BF–BT ceramics when chemical homogeneity is highly required. For instance, improved ferroelectric/piezoelectric activity in BF–BT is usually achieved by the application of quenching as shown in Figure 13a, however, if the chemical heterogeneity in BF–BT-based ceramics is absent or minimal then the improvement in ferroelectric and piezoelectric response is comparable to the quenched ones [131,132]. Realistically, thermal quenching operation in industrial scale is not feasible and could require a new set-up, and therefore chemically homogeneous BF–BT-based ceramics without requiring quenching could be advantageous. On the other hand, it has been reported that chemically and structurally inhomogeneous BF–BT ceramics could enhance the dielectric energy storage density by furnace cooling [132,133]. Hence, the consensus derived from a large number of studies on BF–BT is that the structure and functional properties are highly sensitive to the doping species, doping quantity and heat treatment (cooling procedure), suggesting that there may be more than one route to tune the desired functionality of these ceramics.

Characterization of the chemically and structurally inhomogeneous ceramics requires not only advanced characterization tools but also simultaneous correlation of the microscopic and crystallographic patterns to determine the precise phase assemblages. A recent work of McCartan et al. carried out a correlative approach on donor-doped BF–BT ceramics using transmission electron microscope/scanning transmission electron microscope (TEM/STEM) combining electron energy loss spectroscopy (EELS) for chemical mapping and scanning precession electron diffraction (SPED) patterns for the analysis of octahedral tilting via spotting weak superlattice structures [134]. This method provided unprecedented details within compositionally graded grains, and even leads to the discovery of a barium ferrite type phase within the core-shell structured BF–BT grains, which could be of great interest to multiferroic BF-based solid solution systems.

#### 3.4.3. Heat-Treatment

As mentioned in the previous section, one of the issues hampering the piezoelectric properties in BF-based lead-free piezoelectric ceramics is the high leakage current due to Bi, Fe, and/or oxygen vacancies. In general, defects can be suppressed by controlling the chemical composition through donor or acceptor doping. Recently, a method for reducing the leakage current by controlling the charged defect distribution rather than controlling the number of charged defects using doping or substitution has been proposed [102,135]. The main cause of the decrease in electrical properties due to charged defects is located on the domain wall and forms defect dipoles to interrupt polarization switching [136,137]. Oxygen vacancies help the movement of electrons to generate leakage current.

**Figure 17:**
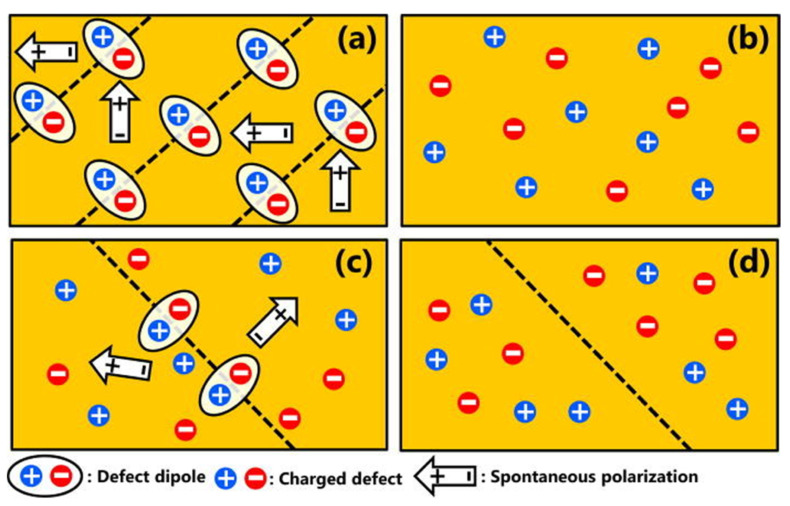
Schematic of defect structure in BiFeO_3_–BaTiO_3_ ceramics; (**a**) as-sintered, (**b**) at high temperature, (**c**) furnace cooling after annealing, and (**d**) quenching after annealing. Schemes follow the same formatting. Reprinted with the permission of [135]. Copyright © 2017 AIP Publishing.

Recently, Kim et al. proposed that charged defect distribution can be controlled by a quenching process [135], as shown in Figure 17. At high temperatures (above *T*_C_), the piezoelectric material possesses a paraelectric phase with a cubic structure, and the ferroelectric domains disappear. Thus, charged defect distributions were homogenously formed, as shown in Figure 17b, and the homogenous charged defect distribution was maintained at room temperature by the quenching process. Therefore, even if the domain is formed again at room temperature with the ferroelectric phase, the charged defects cannot move to the domain walls and are located inside the domain, as shown in Figure 17d. Since the defect dipoles cannot be formed and moved in the domain wall by the quenching process, the leakage current is reduced and the electrical properties are thus enhanced.

**Figure 18:**
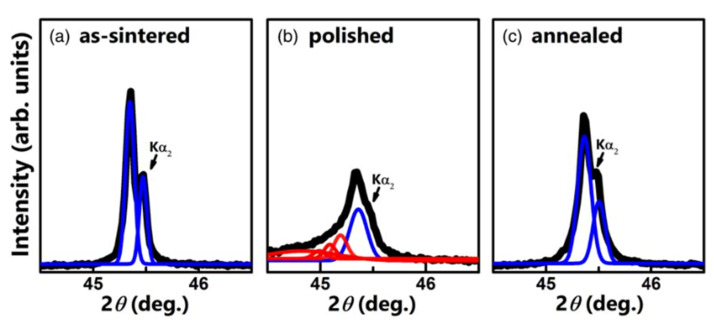
The (200) diffraction peaks of (**a**) as-sintered ceramics with undamaged layers, (**b**) surfaces polished ceramics with damaged and undamaged layers, and (**c**) thermally annealed ceramic’s surfaces. The damaged and undamaged layers on diffraction peaks were identified by profile fitting on Pseudo-Voigt function. Reprinted with the permission of [140]. Copyright (2019) The Japan Society of Applied Physics.

Another problem is that the mechanical stress generated during the ceramic preparation process reduces piezoelectricity and ferroelectricity in lead-free ceramics [138,139]. Mechanical stress causes a decrease in the piezoelectric and ferroelectric properties of lead-free ceramics. Mechanical stress can be induced on ceramic surfaces during polishing and cutting in ceramic processing. Nam et al. suggested that mechanical stress is induced on the surfaces of the samples during cutting and polishing, and damage layers are formed in the BT–BMT–BF ceramics, as confirmed by XRD [140]. The (200) diffraction peak of BT–BMT–BF showed a single peak after sintering, as shown in Figure 18a. It can be observed that a broad shoulder peak occurs on the left side of the single peak after polishing, as shown in Figure 18b, which implies that a damaged layer with a larger lattice constant than the undamaged layer was formed on the surfaces. After annealing, the broad peak was recovered with a single peak, as shown in Figure 18c. This indicates that the damaged layer was recovered by annealing, and as a result, the piezoelectric properties were enhanced. Recently, Wang et al. also raised the similar issue with the distorted surface layers caused by various surface preparation methods in BF–BT ceramics and using grazing incidence XRD was suggested to be an accurate technique for the evaluation of the observed surface effects [141].

Phase decomposition occurs during cooling in a thermodynamically unstable temperature region. Phapale et al. proposed that BF is metastable with respect to Bi_2_Fe_4_O_9_ and Bi_25_FeO_39_ based on high-temperature XRD and isothermal heat-treated XRD data [142]. Recently, Grande et al. reported the decomposition of BF to Bi_25_FeO_39_ and Bi_2_Fe_4_O_9_ in the temperature range of 720–1040 K [143]. The effect of the metastable temperature region on the piezoelectric and ferroelectric properties of the BF-based ceramics was studied by controlling the quenching rate.

**Figure 19:**
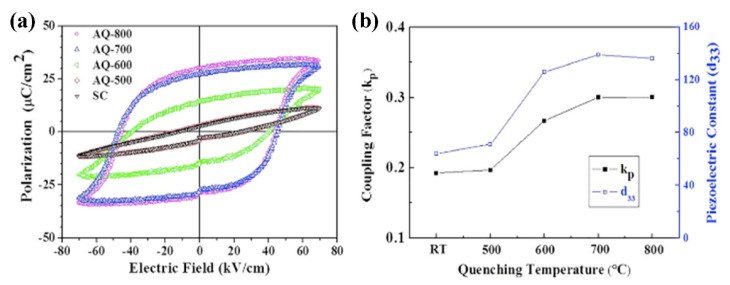
(**a**) *P*-*E* hysteresis loop and (**b**) *d*_33_ and *k*_p_ of 0.75BiFeO_3_–0.25BaTiO_3_ ceramics as a function of quenching temperature. (SC: slowly cooled, AQ−*x*: air-quenching from *x* °C, *x* = 500, 600, 700, and 800). Reprinted with the permission of [101]. Copyright © 2016 AIP Publishing.

Recently, Kim et al. investigated the effect of quenching rate on the piezoelectric and ferroelectric properties in 0.75BT–0.25BF ceramics [101]. The piezoelectric and ferroelectric properties were enhanced in the BF–BT ceramics when quenching at 800 °C was performed without going through the temperature at which decomposition occurred, as shown in Figure 19. A similar result was obtained for the BT–BMT–BF system [102]. Nam et al. investigated the quenching temperature effect on piezoelectric properties in BT–BMT–BF system. Quenching was conducted the various temperatures, and the piezoelectric property of BT–BMT–BF ceramics was dramatically enhanced when quenching at 800 °C.

#### 3.4.4. Pseudo-Cubic Structure

**Figure 20:**
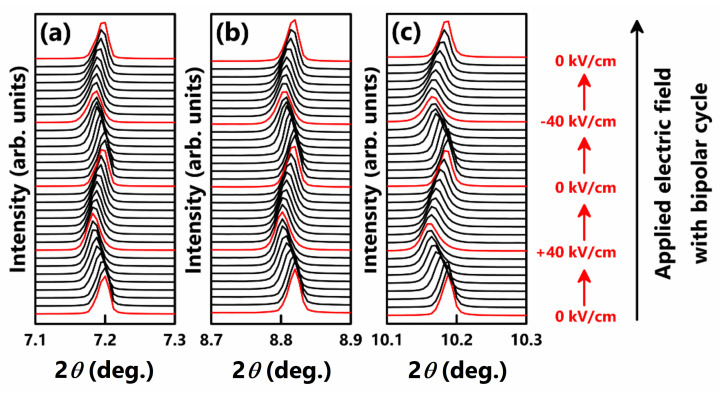
In-situ synchrotron X-ray diffraction of 0.67BiFeO_3_–0.33BaTiO_3_ ceramics under the AC cycling from −40 kV/cm to +40 kV/cm with selected angle for (**a**) (110) peak, (**b**) (111) peak, and (**c**) (200) peak. Reprinted with the permission of [144]. Copyright © 2021 Journal of the Ceramic Society of Japan.

As mentioned in Section 3.1, the determination and understanding of pseudo-cubic structure is an issue in BF-based lead-free piezoelectric ceramics. The crystal structure of ceramics that seem to have a cubic-like structure with non-polar centrosymmetry and a small lattice distortion is often called a pseudo-cubic structure. Various BiFeO_3_-based lead-free piezoelectric ceramics (e.g., BF–BT, and BT–BMT–BF) exhibit piezoelectricity and ferroelectricity in pseudo-cubic structures [144,145]. To understand the pseudo-cubic structure, it is important to monitor the crystal structure under an applied electric field. Structural stabilization under an electric field is key to elucidate the origin of piezoelectricity in a pseudo-cubic structure. In a BNT-based lead-free system, the crystal structure was investigated under an applied electric field, and the pseudo-cubic structure changed to a tetragonal structure under an electric field, which is called an electric-field-induced phase transition [146,147]. Recently, Kim et al. and Fujii et al. investigated the phase transition in a BF-based lead-free system under an electric field using in-situ synchrotron radiation XRD [144,145]. The single peaks that are characteristics of a cubic structure were detected without an electric field, whereas the ceramics exhibited significantly large piezoelectricity and ferroelectricity. The single peaks only showed significant shifts under the electric field in the BF–BT system and was maintained their shape under an applied electric field, as shown in Figure 20. Thus, it was evident that electric-field-induced phase transition did not occur in the BF–BT system.

**Figure 21:**
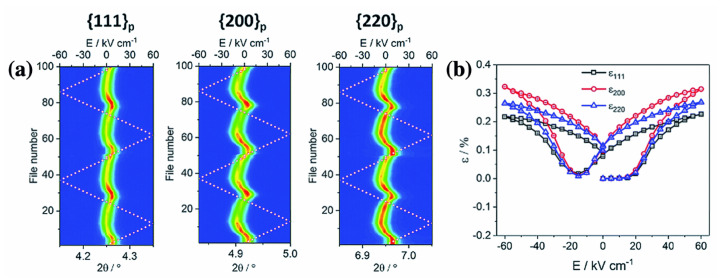
(**a**) 2D contour plot of (111), (200), and (220) peak profiles in 0.01Nd(Li_0.5_Nb_0.5_)O_3_-doped BF–0.30BT under the electric field. (**b**) Lattice strain anisotropic from (111), (200), and (220) peaks, respectively. Reproduced from [149] with permission from the Royal Society of Chemistry.

Various models have been suggested to understand piezoelectricity and ferroelectricity in pseudo-cubic structures in the lead-free system [148,149,150,151,152]. Wang et al. suggested the existence of multiple symmetric polar nanoregions in the Nd(Li_0.5_Nb_0.5_)O_3_-doped BF–0.30BT system [149]. A large strain of ~0.6% was found in 0.01Nd(Li_0.5_Nb_0.5_)O_3_-doped BF–0.30BT ceramics at 150 kV/cm in in-situ poling synchrotron XRD. The pseudo-cubic structure was maintained without changing the full width at half maximum (FWHM) of the XRD peak before and after the application of the electric field. However, the strain anisotropy in the XRD peak (200 > 220 > 111), as shown in Figure 21, indicates the possibility of the presence of multiple symmetrically polar nano regions that allow for a large average distortion in the applied electric field direction. Similar properties and deformation behaviors have also been reported for several BF–BT-based systems. The polar nano regions might have been caused by the asymmetry of the local structure. Therefore, it is important to investigate the local structure at the atomic level to understand the mechanism of the pseudo-cubic structure.

**Figure 22:**
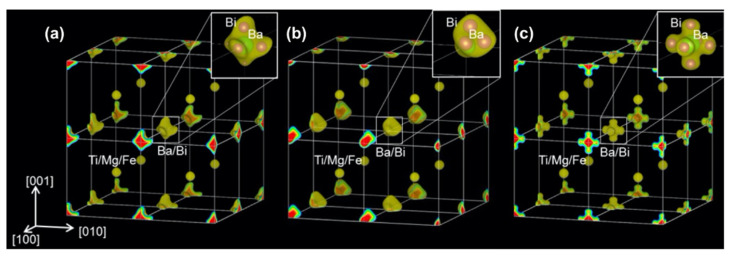
Electron density distribution of 0.30BaTiO_3_–0.10Bi(Mg_1/2_Ti_1/2_)O_3_–0.60BiFeO_3_ at (**a**) 300 K, (**b**) 600 K, and (**c**) 900 K. Reprinted with the permission of [152]. Copyright © 2021 Springer Nature.

Recently, Kim et al. investigated the crystal structure in the BF–BT system and found that the Bi ion was disordered at the *A*-site in the BF–BT system [152]. Kuroiwa et al. provided direct evidence of Bi ion’s disordering in a BMT-doped BF–BT system by electron charge density distribution, as shown in Figure 22 [151]. The electron density distribution around the *A*-site was distinguished from that around the *B*-site. The electron density distribution around the *A*-site (Ba/Bi) is anisotropic in the <100> direction, whereas the *B*-site is isotropic, which indicates that the *B*-site ion (Mg/Ti/Fe) has the same atomic position and the *A*-site ion (Ba/Bi) has a different atomic position. The Bi ion was disordered with the 6-site along the <100> direction in the paraelectric phase at 900 K with a cubic structure, and in the ferroelectric phase at 300 K with a pseudo-cubic structure, as shown by the enlarged electron density distribution at the *A*-site in Figure 22a; meanwhile, with the 3-site, it was disordered by a large rhombohedral distortion at 600 K. The Bi ion disordering was changed by the crystal structure in the BMT-doped BF–BT system.

**Figure 23:**
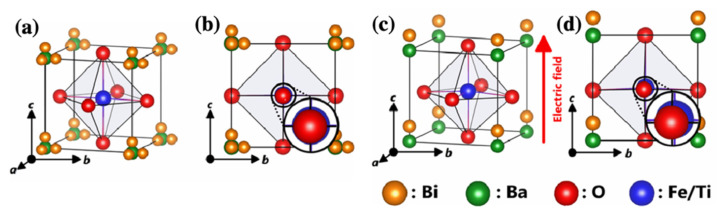
Fluctuated Bi ion’s ordering model under the electric field; (**a**) unit-cell of 0.67BiFeO_3_–0.33BaTiO_3_ and (**b**) (100) view of crystal structure without electric field. (**c**) Bi ion’s partial ordering model and (**d**) (100) view of crystal structure under the applied electric field along [001] direction. Reprinted with the permission of [152]. Copyright © 2021 Elsevier.

The Bi ion off-centered from the *A*-site plays an important role in piezoelectricity. The Bi ion disordering is a vital aspect in the BF–BT and BT–BMT–BF systems. The Bi ion is disordered in the BF–BT system without electric field, as shown in Figure 23a,b. The off-centered Bi ions were ordered along the direction of the electric field. For example, the Bi ion can be ordered onsite when an electric field is applied along the [001] direction, as shown in Figure 23c,d. The change in the position of the Bi ion from disordered to ordered under an applied electric field is similar to ferroelectric domain switching [151,152]. Bi ions aligned in the direction of the electric field could be switched in the opposite direction by changing the direction of the electric field. Therefore, the ordering of Bi ions can induce piezoelectricity and ferroelectricity (even in a pseudo-cubic structure).

## 4. Summary

In terms of sustainability and environmental problems, lead-free materials are expected to be key materials in the future. However, the properties of lead-free materials are inferior to those of lead-based materials. The lead-free BF-based systems are a relatively new research area compared to BT-based, KNN-based, and BNT-based systems. The significantly large piezoelectric properties and high phase transition temperatures of BF-based systems imply their potential for high-temperature applications. Nevertheless, the BF-based lead-free systems still have several controversies, e.g., the high conductivity is a major issue in BF-based systems. Volatilization of Bi^3+^ during the high-temperature sintering process and fluctuation of multivalent Fe (Fe^2+^, Fe^3+^and Fe^4+^) could cause high leakage current. Therefore, a compositional design that can precisely control such causes is required to overcome high conductivity in BF-based systems.

The impact of chemical heterogeneity on the functional and (micro)structural properties of BF-based ceramics has been reviewed in light of recent findings.

Another important aspect is related to the role of pseudo-cubic structure. BF–BT-based materials exhibit large variations in functional properties depending on the crystal structure. In particular, it has been reported that the piezoelectric properties increase when a pseudo-cubic structure is present. Recently, efforts have been made to understand the mechanism of the pseudo-cubic structure in BF-based systems using various advanced analytical techniques, such as in-situ XRD and in-situ transmission electron microscopy (TEM). The results show that the underlying mechanism is attributed to Bi ordering. More precise techniques, however, are required to resolve their exact chemical-crystallographic structures.

**Figure 24:**
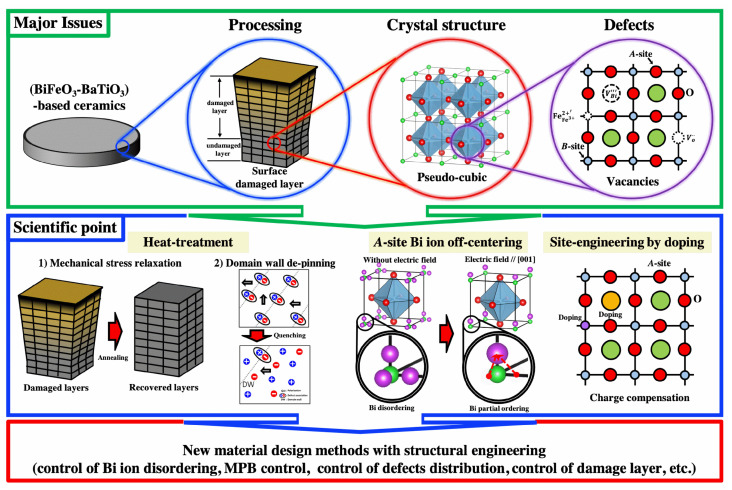
Summary of the major issues and scientific viewpoints in BF-based lead-free piezoelectric ceramics.

Briefly, the major issues of the BF–BT-based system can be summarized in view point of processing, crystal structure, and defects, as shown in Figure 24. These issues have been explained by the damage layer relaxation or control of charged defect distribution by heat-treatment, Bi ion partial ordering under the electric field, or control of high conductivity by doping. Various mechanisms or processing methods have been suggested from a scientific viewpoint to clarify major issues in BF–BT-based systems. However, BF-based lead-free system still should clarify its unclear local structure and related mechanism. Determining the accurate crystal structure and related mechanisms is crucial for developing new material design methods, such as structural engineering (MPB control, control of the disordered Bi ion, etc.), and will contribute to the development of next-generation piezoelectric ceramics. Recently, research on magnetic properties, energy harvesting, and dielectric energy storage properties has also been conducted on BF-based systems for various applications. Research on various potential properties and controversial issues in BF-based materials has led to an in-depth understanding of lead-free piezoelectric materials and accelerated the search for next-generation electroactive materials.

## Data Availability

Not applicable.
